# Effects of Mixture of Pharmaceuticals on Early Life Stages of Tench (*Tinca tinca*)

**DOI:** 10.1155/2014/253468

**Published:** 2014-03-20

**Authors:** Vlasta Stancova, Lucie Plhalova, Marta Bartoskova, Dana Zivna, Miroslav Prokes, Petr Marsalek, Jana Blahova, Misa Skoric, Zdenka Svobodova

**Affiliations:** ^1^Department of Veterinary Public Health and Animal Welfare, University of Veterinary and Pharmaceutical Sciences Brno, Palackeho tr. 1/3, 61242 Brno, Czech Republic; ^2^Institute of Vertebrate Biology, Academy of Sciences of the Czech Republic, v.v.i., Kvetna 8, 603 65 Brno, Czech Republic; ^3^Department of Pathological Morphology and Parasitology, University of Veterinary and Pharmaceutical Sciences Brno, Palackeho tr. 1/3, 612 42 Brno, Czech Republic

## Abstract

Ubiquitous occurrence of pharmaceuticals in aquatic environment results in concern about potential adverse the effects on nontarget organisms. In water, drugs are present in complex mixtures, in which complicated interactions affect toxicity of single components. The purpose of this study was to examine effect of 35-day-long exposure to mixture of ibuprofen, diclofenac, and carbamazepine on the mortality, growth, early ontogeny, and histopathological changes in tench (*Tinca tinca*). Early life stage toxicity test was carried out using a modified protocol according to OECD guideline 210. Exposure to mixture of pharmaceuticals at concentration of 60 **μ**g*·*L^−1^ for each substance was associated with significant increase in mortality, as well as significant increase in growth and elevated incidence of malformations. Any of the tested concentrations resulted in histopathological changes of liver, kidney, skin, or gill. After fourteen days of exposure there was short-term delay of development related to increased concentrations of pharmaceuticals in the mixture (2, 20, and 60 **μ**g*·*L^−1^). Environmentally relevant concentrations (0.02; and 0.2 **μ**g*·*L^−1^) used in this experiment did not result in toxic impairment of tench.

## 1. Introduction

Human and veterinary pharmaceuticals and their metabolites belong due to their overproduction and indiscriminate usage to ubiquitous environmental contaminants present in the ground and surface waters at a magnitude of ng·L^−1^ to *μ*g·L^−1^ [1]. In effluents of three Swiss wastewater treatment plants (WWTP), the concentrations of pharmaceuticals reached 1.3 *μ*g·L^−1^ for ibuprofen, 0.99 *μ*g·L^−1^ for diclofenac, and 0.95 *μ*g·L^−1^ for carbamazepine [2]. The study of French scientists [3] demonstrates that concentration of pharmaceuticals in surface water is two or three times higher than in treated drinking water.

Pharmaceuticals can be excreted, primarily via urine and faeces, either as an unchanged parent compound or in the form of metabolites or as conjugates of glucuronic and sulphuric acid, and they can then enter aquatic ecosystems via different ways [4]. Moreover, conventional technology used in wastewater treatment plants appears as insufficient to remove these specific compounds [5]. Some substances (e.g., carbamazepine) are not removed during wastewater treatment. Carbamazepine passes the plants without any reduction and effluent concentrations are in the range of influent concentrations [6]. However, removal efficiencies for ibuprofen and diclofenac are high 65–100 and 30–100%, respectively; they are still ubiquitous and are present at considerable concentrations in river waters [7–10]. This could be due to the fact that their concentrations in the inlets are so high that the remains in the effluents are still significant [11]. Carbamazepine is relatively lipophilic, with an octanol/water partition coefficient of 2.2, and is consistently found at relatively high concentrations in aquatic environment. This compound was proposed as a suitable anthropogenic marker of urban effluents [12]. Information about concentrations of ibuprofen, diclofenac, and carbamazepine in surface water is summarized in Table 1. Detection of pharmaceuticals in the environment has resulted in concern for potential adverse effects on nontarget species.

Experiment by Loos et al. [14] showed that the polar pharmaceuticals (ibuprofen and diclofenac) are slowly degraded in water (by a factor of around 20% after 3 weeks). Photodegradation half-life time of diclofenac is much faster than carbamazepine and humic acids (concentration of 5.0 mg·L^−1^) act as inner filters during the photodegradation of carbamazepine and diclofenac [17].

The wide spectrum of substances detected in receiving river waters indicates that WWTP outlets are major contributors to pharmaceuticals in the aquatic environment [11]. Although most of these compounds are present at low concentrations, many of them raise considerable toxicological and ecotoxicological concerns, particularly when present as components of complex mixtures. It is very difficult to assess the effect on the aquatic environment of the thousands of synthetic and natural trace contaminants that may be present in water at low concentrations [18].

Pharmaceuticals are designed to have specific mode of action and many of them for persistence in the organism. According to Láng and Köhidai [19], toxicity of diclofenac based on the proliferation inhibition of ciliate* Tetrahymena pyriformis* is higher than toxicity of ibuprofen. The reported order of toxicity diclofenac > ibuprofen was confirmed by Cleuvers [20] too. Although detected environmental concentrations of pharmaceuticals are often low, these compounds are present in mixtures [21]. Substances applied at no effect concentration can contribute to the total mixture effect which in turn can become significant. Concentration addition was observed, for example, for NSAIDs (nonsteroidal anti-inflammatory drugs) in Daphnia and algal bioassays [20]. For pharmaceuticals chronic exposure is much more relevant than acute exposure.

The aim of this study was to assess subchronic toxic effects of mixture of drugs (ibuprofen, diclofenac, and carbamazepine) on tench (*Tinca tinca*). Our work mainly focused on the growth parameters, histopathology, early ontogeny, incidence of malformations and mortality of embryos, and larval stages in tench affected by mixture of pharmaceuticals.

## 2. Materials and Methods

### 2.1. Experimental Protocol

Our laboratory experiment was carried out using test solutions containing mixture of ibuprofen (IBU), diclofenac sodium salt (DCF), and carbamazepine (CBZ) as test substances (Table 2). All test substances were purchased from Sigma-Aldrich Co.

Table 3 summarizes various physical properties of IBU, DCF, and CBZ, including the dissociation constant (p*K*_a_), octanol-water partition coefficient (log *K*_ow_) [21], and the percentage of parent compound excreted from the human body [22].

Embryo-larval toxicity test was carried out using a modified protocol according to OECD guideline 210 (fish, early life stage toxicity test) [23]. Fertilized eggs of tench were obtained from a commercial fish farm.

Experiment took place in 500 mL crystallization dishes, each containing 100 randomly distributed embryos (24 hours after fertilization). The experiment was conducted in triplicate (a total of 300 fertilized eggs for each experimental group and each control). The embryos and larvae of tench were exposed to the mixtures of pharmaceuticals on nominal concentrations of 0.02, 0.2, 2, 20, and 60 *μ*g·L^−1^ (for each pharmaceutical), and experimental groups were named M1, M2, M3, M4, and M5, respectively. Duration of the test was 35 days. The two lowest used concentrations simulated environmental conditions, the third concentration approaches concentration of pharmaceuticals in waste water, and the highest concentration is up to 3000 higher than environmental concentration. Control fish were kept in tap water. Due to use of ibuprofen, diclofenac, and carbamazepine, as test substances, which require the use of dimethylsulfoxide (DMSO) as a solvent, additional control fish were exposed to 0.01% DMSO as a solvent control. The concentration of DMSO in the solvent control corresponded to the DMSO concentration present in the test water containing the highest drug concentration (M5 group). A concentration of 0.01 mL·L^−1^ of DMSO did not result in lethal effects, abnormalities, or changes in growth parameters during an embryo-larval toxicity test on carp [24].

A semistatic method was used, in which solutions of drugs were replaced twice a day. Concentrations of pharmaceuticals in test waters were determined once a week by HPLC. None of the pharmaceuticals used in this study were detected in the dilution water during the study period. During the test, concentrations of pharmaceuticals did not fall below 80% of the nominal concentration. Dead embryos and larvae were removed from crystallization dishes twice a day. Feeding was initiated on day 7. Larvae were fed with freshly hatched* Artemia salina* twice a day* ad libitum *after the bath exchange. Hatching, survival, temperature, pH, and oxygen saturation were recorded daily. The water temperature and pH ranged from 21 to 23°C and 8.4 to 8.8, respectively. A photoperiod consisted of 12 h light/12 h dark segments for each day. Concentration of dissolved oxygen did not fall below 80%.

During the test, larvae were sampled to investigate developmental changes, morphological abnormalities, length, weight, and Fulton's condition factor (FCF). Samples from each concentration and each control were collected on day 7 (after feeding initiated), and on days 14, 21, 29, and 35. All collected fish were fixed in 4% formalin. Ending of the experiment was at the 35th day, when fish were euthanized by carbon dioxide.

### 2.2. Determination of Developmental Stages

The developmental stages of tench were determined according to Peňáz et al. [25–27] who described ontogenesis in tench and common carp, both belong to the same cyprinid family (Cyprinidae). Peňáz distinguished nine embryonic (E1–E9), six larval (L1–L6), and two juvenile (J1-J2) stages.

### 2.3. Length, Weight, and Growth Evaluation

Total length (TL) from the mouth to caudal peduncle was measured stereomicroscopically using a micrometer to be 0.01 mm. Whole body wet weight (*W*) was measured to be 0.1 mg. Fulton's condition factor (FCF) is widely used in fisheries and general fish biology studies. This factor is calculated from the relationship between the weight of a fish in grams and its length in millimeters, with the intention of describing the condition of that individual [28]. In our study FCF was calculated for each sampling time and for each experimental group. Consider

(1) $$\begin{array}{c} \text{FCF}=\frac{W\times 10^{5}}{{\text{TL}}^{3}}. \end{array}$$

The specific growth rate (SGR) is a measure of the percentage body weight increase per day. The mean SGR was calculated for each experimental group in the beginning on day 7 (the first sampling time) and on day 35 (the last sampling time). The SGR was calculated in the following formula:

(2) $$\begin{array}{c} \text{SGR}\left(\%\right)=\frac{\bar{\ln W_{2}}-\bar{\ln W_{1}}}{T_{2}-T_{1}}\times 100, \end{array}$$

where ln is natural logarithm. *W*_1_ is weight of one fish at time *T*_1_ in grams; *W*_2_ is weight of one fish at time *T*_2_ in grams. *T*_1_ is first sampling time; *T*_2_ is last sampling time.

The inhibition of specific growth rate (*I*) for each experimental group was calculated as follows:

(3) $$\begin{array}{c} I\left(\%\right)=\frac{\text{SGR}\;\left(\text{control}\right)-\text{SGR}\;\left(\text{group}\right)}{\text{SGR}\;\left(\text{control}\right)}\times 100. \end{array}$$

### 2.4. Histopathological Examination

The fish were prepared for histopathological examination, fixed in buffered 10% neutral formalin, dehydrated, embedded in paraffin wax, sectioned (cross-section) on a microtome at 4 *μ*m, and stained with hematoxylin and eosin (H&E). The histology of skin, gill, kidney, and liver was examined by light microscopy.

### 2.5. Determination of Ibuprofen, Diclofenac, and Carbamazepine

Measurement of diclofenac, ibuprofen, and carbamazepine in water samples was performed by high-performance liquid chromatography coupled with triple quadrupole tandem mass spectrometry (LC-MS/MS). Sample preparation was based on solid phase extraction. SPEC C_18_ AR cartridges (3 mL, 30 mg, Varian, Inc., Palo Alto, CA, USA) were used. One milliliter of the sample was passed through a preconditioned cartridge (500 *μ*L methanol and 500 *μ*L water). The analyte was eluted with 1 mL acetonitrile and used for LC-MS/MS analysis. A Thermo Scientific UHPLC Accela 1250 system was connected to a Thermo Scientific TSQ Quantum Access MAX triple quadrupole instrument (Thermo, San Jose, CA, USA) equipped with heated electrospray ionization (HESI-II) probe. A Thermo Scientific Hypersil C_18_ (2.1 mm × 50 mm, 1.9 *μ*m) column was used at a constant flow rate of 300 *μ*L·min^−1^ by an isocratic elution method with acetonitrile/water 70/30 (v/v). The full loop injection volume of the sample was set at 20 *μ*L. The heated electrospray ionization was operated in the positive-ion mode for carbamazepine and in the negative-ion mode for ibuprofen and diclofenac under the following conditions: capillary temperature: 325.0°C; vaporizer temperature 300.0°C; sheath gas pressure 35.0 psi; auxiliary (drying) gas 10 a.u.; spray voltage 3300 V (−3300 V for ibuprofen and diclofenac). Standards were purchased from Sigma-Aldrich (St. Louis, MO, USA). All solvents were residual analysis purity (Chromservis s.r.o., CZ). For our QA/QC program, the instrument was calibrated daily with multilevel calibration curves. Procedural blank and solvent blank were analyzed for every set of 20 samples. The spiked recoveries were 97% for ibuprofen, 99% for diclofenac, and 99% for carbamazepine. Reported concentrations are after corrections based on the recoveries. Coefficients of variation for between-series were 4.1% for ibuprofen, 3.5% for diclofenac, and 2.9% for carbamazepine. The limits of detection were determined as 3 : 1 signal versus noise value (S/N) and were 9 ng·L^−1^ for ibuprofen, 7 ng·L^−1^ for diclofenac, and 5 ng·L^−1^ for carbamazepine.

### 2.6. Statistical Analysis

Growth parameters were tested using statistical analysis performed using software Unistat 5.6 for Excel. Data were evaluated for normal distribution by Shapiro-Wilk test. Normal distributed data were compared using parametric ANOVA. Since data were not normally distributed, significance of differences between control, control-solvent group, and experimental groups was tested by nonparametric Kruskal-Wallis followed by multiple comparisons. Mortality was evaluated using contingent tables. Levels of significance were set to *P* < 0.001 highly significant (**) and *P* < 0.01 significant (*).

## 3. Results 

### 3.1. Exposure Concentrations

See Table 4.

### 3.2. Hatching

There was not any observed negative effect of any concentration of mixture of drugs on the hatching. The hatching began in both control groups and in all experimental groups on the second day of the experiment and was completely finished on the fourth day. Hatching success was at least 95% for both control groups and all experimental groups.

### 3.3. Mortality

**Figure 1:**
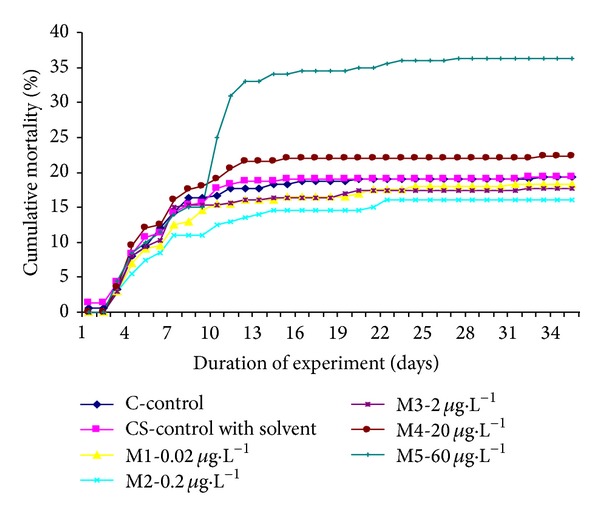
Cumulative mortality in controls and experimental groups affected by mixture of ibuprofen, diclofenac, and carbamazepine, during 35-day-long test.

Mortality at the end of the experiment was 19.3, 19.3, 18.5, 16.0, 16.7, 22.3, and 36.3% in C, CS, M1, M2, M3, M4, and M5, respectively. Significant (*P* < 0.001) increase in mortality between control and M5 groups has been found. Differences between mortality in CS, M1, M2, M3, and M4 in comparison with C group were not significant. Cumulative mortality is depicted in Figure 1.

### 3.4. Length and Weight Parameters, Condition, and Growth Rate

Growth parameters did not differ between control and control-solvent groups. Statistically significant (*P* < 0.01) effect of the highest tested concentration of drugs mixture (M5) on weight of fish on the 7th and 14th days and high significant (*P* < 0.001) effect on weight on the 35th day of the test have been found. Effect on total length of fish of groups M4 (*P* < 0.01) and M5 (*P* < 0.001) after seven-day-long exposure to pharmaceuticals has been found. After fourteen days, statistical significance on decrease of length (*P* < 0.01) of fish in experimental group M5 has been found. Highly significant increase of length (*P* < 0.001) of fish of M5 group occurred after 35-day-long exposure. Length and weight parameters are depicted in Table 5.

After 35 days of experiment, significantly higher (*P* < 0.001) FCF has been found in tench exposed to M5 than in tench from control group (Table 6).

### 3.5. The Occurrence of Malformations

**Figure 2:**
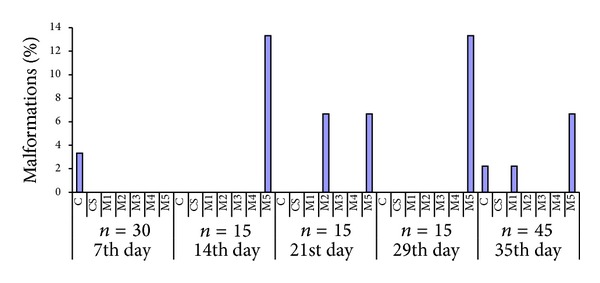
Percentage of malformations found in C-control, CS-control with solvent, M1, M2, M3, M4, and M5—mixture of ibuprofen, diclofenac, and carbamazepine on concentration of 0.02, 0.2, 2, 20, and 60 *μ*g·L^−1^, respectively, after 7, 14, 21, 29, and 35 days of exposure.

At first sampling on the seventh day of the experiment, shortened body and curved tail have been found in control group. The same kind of malformations was found in group M5 (*c* = 60 *μ*g·L^−1^) at second sampling on the fourteenth day. Deformations of eyes and lower jaws in groups M2 (*c* = 0.2 *μ*g·L^−1^) and M5 were discovered at the third sampling. Only in M5 group there were uncovered deformations of eyes and lower jaws at the fourth sampling. In samples from the 35th day, malformations in control and M1 (0.02 *μ*g·L^−1^) and M5 group were found. Most common were ocular malformations (lack of eye and pigment-deficient eye) and lower jaws defects. Alimentary canal defects and scoliosis occurred rarely. The percentage of malformations is depicted in Figure 2.

### 3.6. Histopathology

Histopathological examination of liver, kidney, gill, and skin of tench in both control groups and experimental groups (C, CS, M1, M2, M3, M4, and M5) showed no pathomorphological changes.

### 3.7. Early Ontogeny

**Figure 3:**
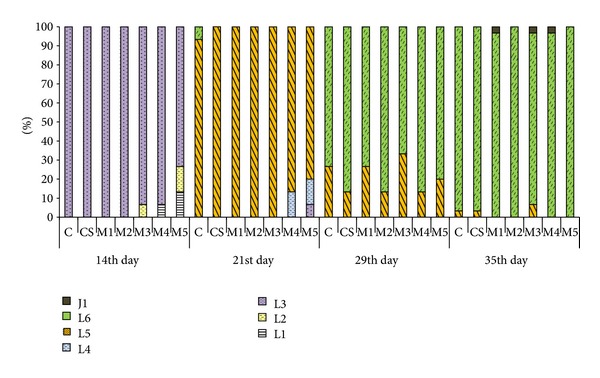
Representation of development stages in experimental groups C-control, CS-control with solvent, M1, M2, M3, M4, and M5—mixture of ibuprofen, diclofenac, and carbamazepine concentration of 0.02, 0.2, 2, 20, and 60 *μ*g·L^−1^, respectively, on the 14th, 21st, 29th, and 35th day of experiment.

At first sampling on the seventh day, all fish in all experimental groups were L1 larval stage. After 14 days of exposure, all fish from C, CS, M1, and M2 groups belonged to L3 stage. In M3 and M4, almost 7% of fish were determined as L1 and L2 stages, respectively. M5 group consisted of L3 (majority) and more than 13% were L1 and L2 stages. After 21 days of exposure, most fish were in L5 stage. In C, M4, and M5, L6, L4, and L3 also occurred, respectively. Fish from both control groups and all experimental groups belong to L6 and L5 stages after 29-days-long exposure. At the end of the test (the 35th day of exposure) most fish were determined in larval L6 stage and some fish were determined in stage L5 and some already achieved to be in first juvenile stage-J1 (Figure 3).

### 3.8. Behavior

All fish in both control groups and experimental groups (M1, M2, M3, M4, and M5) showed normal behavior.

## 4. Discussion

The aquatic environment is increasingly exposed to complex mixtures of pollutants. A chemical can be more toxic when it is mixed with other chemicals, because of chemical interactions commonly referred to as cocktail effects. Mixed exposure can result in interactions, which means that one chemical affects absorption, distribution, metabolism, or excretion of another chemical [29]. The predominant interaction type observed in mixtures of pharmaceuticals (IBU, DCF, propranolol, and metoprolol) was antagonism, and the frequency of its detection increased in general with the mixture concentration. The predominance of antagonism in the higher concentration range might be explained by a potential competitive inhibition between two pharmaceuticals acting in the same way. Additivity was observed only in the 37% of the mixtures and synergism was the rarest type of interaction that was obtained only in 4% of the cases [19]. Mixture toxicity of the compounds could be accurately predicted using the concept of concentration addition. Evaluation of the ecotoxicity of DCF, IBU, naproxen, and acetylsalicylic acid using acute* Daphnia* and algal tests showed that toxicity of the mixture was considerable, even at concentrations at which the single substances showed no or only very slight effects, with some deviations in the* Daphnia* test [30].

In our experiment, hatching success was not disrupted by mixture of IBU, DCF, and CBZ up to concentration of 60 *μ*g·L^−1^. Similarly, embryonic exposure to mixture of CBZ, acetaminophen, gemfibrozil, and venlafaxine at concentrations of 0.5 and 10 *μ*g·L^−1^ or diluted wastewater treatment effluent (5% and 25%) did not affect hatching success of zebrafish (*Danio rerio*) [31]. In hatching, there were some differences between vulnerability of fish species. While there was recorded delay in hatching time after exposure of zebrafish embryos to DCF at 1, 1.5, and 2 mg·L^−1^ [32, 33], no negative effects of DCF up to concentration of 3 mg·L^−1^ on the hatching and viability of carp embryos were found [34]. Consequences of long-term exposure to DCF up to 3 months were evaluated in a freshwater medaka fish (*Oryzias latipes).* Exposure to 0.001–10 mg·L^−1^ resulted in significant decreasing trend in hatching success and delay in hatch. The hatching of the eggs produced from the fish exposed to 10 mg·L^−1^ was completely interfered [35]. Parental exposure to as low as 0.1 *μ*g·L^−1^ IBU in* Oryzias latipes* delayed hatching of eggs even when they were transferred to and cultured in clean water. Delayed hatching is environmentally relevant because this may increase the risk of being predated [36]. Nanoinjection of DCF and CBZ into medaka embryos clearly decreased their hatchability and some doses delayed the hatching time [37]. Memmert et al. [38] conducted early life stage test with rainbow trout and zebrafish. Fish were affected by DCF in concentration range: 3.2–1000 *μ*g·L^−1^. Hatching rate at all tested concentrations did not differ significantly from the control in both fish species.

While mortality in M1, M2, M3, and M4 groups was comparable with control, situation in group M5 at the end of our experiment was different and mortality increase was significant (*P* < 0.001). Early life stage parameters such as egg and embryo mortality did not show significant differences in comparison with control group after exposure to diclofenac (1–2000 *μ*g·L^−1^) and its solvent DMSO [32]. Embryo mortality was elevated with exposure to 10 *μ*g·L^−1^ of mixture of CBZ, acetaminophen, gemfibrozil, and venlafaxine [38]. The effect of IBU on medaka increased with duration of exposure. Survival of adult fish (120 dph—days after hatching) exposed to IBU as little as 1 *μ*g·L^−1^ was significantly less than in the controls, while survival of fry (7 dph) was not affected even at the maximum test concentration of 1000 *μ*g·L^−1^ [36]. There were no significant changes in survival for CBZ and IBU (up to 1000 *μ*g·L^−1^) in fathead minnow at 28 days after hatching [39].

The rate at which fish grow depends on a number of factors including species, age, genetic potential, water temperature, health, and quantity and quality of food. Mixture of drugs used in this experiment at concentrations of 0.02, 0.2, 2, and 20 *μ*g·L^−1^ did not have any effect on the growth parameters of tench after 35 days of exposure, but the concentration of 60 *μ*g·L^−1^ affected weight and length of tench significantly. Surprisingly, fish growth was not retarded but boosted. Furthermore, FCF of tench exposed to 60 *μ*g·L^−1^ mixture of IBU, DCF, and CBZ increased more significantly than control fish after 35 days. However, there was the highest (*P* < 0.001) mortality in experimental group M5 and amount of fish in aquaria could also affect fish growth. Exposure to amiodarone and clozapine in early life stage toxicity test on fathead minnow (*Pimephales promelas*) also resulted in a significant increase in growth at concentrations of 1020 and 30.8 *μ*g·L^−1^, respectively [39]. Generally, stress conditions such as polluted aquatic environment result in fish growth decrease. Stepanova et al. [34] studied impact of DCF in early life stages of common carp after 30 days of exposure. They did not find any effect on the growth up to concentration of 3 mg·L^−1^. After 144 days of exposure to IBU (0.01–1000 *μ*g·L^−1^), the length, weight, and condition factors of surviving adults of medaka were not affected [36]. Generally, stress conditions such as polluted aquatic environment result in fish growth decrease. Retarded growth of zebrafish in concentrations of DCF above 1.5 mg·L^−1^ [33] was recorded. Neither length nor weight of rainbow trout was affected by DCF up to 1000 *μ*g·L^−1^ [38]. CBZ and IBU did not result in any significant changes in fathead minnow growth at concentrations of 62.5, 125, 250, 500, and 1000 *μ*g·L^−1^ [39]. Chronic toxic effects of CBZ on rainbow trout were investigated. Fish were exposed to sublethal concentrations for 42 days. Compared with the control, there was a significantly lower (*P* < 0.05) FCF in fish exposed to the highest concentration of 2.0 mg·L^−1^, but FCF in fish exposed to 1.0 *μ*g·L^−1^ and 0.2 mg·L^−1^ were not changed significantly [40].

The early stages of embryonic development in fish generally exhibit a high incidence of malformations. These commonly include deformities of head and spinal column, ocular deformities, or yolk-sac resorption abnormalities. The natural background level of embryonic malformations is generally expected to be less than 10% [41]. In our experiment we recorded almost 14% of malformations in M5 group at the 14th and 29th days of exposure. We suppose that such occurrence of malformations is consequence of exposure to high concentration (60 *μ*g·L^−1^) of pharmaceuticals. Most common were ocular malformations and lower jaws defects. Alimentary canal defects and scoliosis occurred rarely. Exposure to DCF (more than 1.5 mg·L^−1^) resulted in yolk-sac and tail deformations of zebrafish [33]. Exposure of embryos to mixture of CBZ, acetaminophen, gemfibrozil, venlafaxine (10 *μ*g·L^−1^), or diluted wastewater treatment effluent (25%) significantly increased the incidence of developmental abnormalities. This increase was primarily caused by an increase in the occurrence of yolk-sac edema [38].* In ovo *nanoinjection of diclofenac (12 ng DCF egg^−1^) and carbamazepine (12 ng CBZ egg^−1^) caused significant impairments of embryonic development [37]. Early developmental stages of fathead minnow fish exposed to mixture of drugs (DCF, triclosan, naproxen, gemfibrozil, IBU, salicylic acid, and acetaminophen) at concentrations of 100 and 300 ng·L^−1^ showed a significant increase in yolk-sac abnormalities, eye deformities and hemorrhaged embryos, and spinal deformities in comparison with control [42]. It seems that early life stages of fish are particularly vulnerable to damage caused by pharmaceuticals.

Study of chronic histopathological effects of DCF on rainbow trout (*Oncorhynchus mykiss*) revealed that 28 days of exposure resulted in renal lesions and alterations to the gills at a concentration of 5 *μ*g·L^−1^ [43]. The lowest observed effect concentration (LOEC) of 1 *μ*g·L^−1^ for induction of cytological alterations in liver, kidney, and gills in rainbow trout [44] was reported. Similar results have been found in brown trout (*Salmo trutta *f.* fario*). Exposure to DCF in concentration ranges commonly found in the environment can result in adverse effects in various organs, especially kidney or gill [45]. Adverse effects of DCF on kidney and villi in the intestine from concentration of 1 *μ*g·L^−1^ after 21 days of exposure in rainbow trout were recorded [46]. CBZ at 0.5 and 10 *μ*g·L^−1^ caused altered ovarian histology in female zebrafish. Six-week -long exposure to CBZ also significantly altered kidney proximal tubule morphology but did not change liver histology [47]. Upper described impacts of single pharmaceutical to histopathology of fish organs are in contrast with our findings, where either concentration of 60 *μ*g·L^−1^ of mixture of IBU, DCF, and CBZ did not cause any histopathological changes in liver, kidney, skin, or gill. Our results are in compliance with many other studies [34, 36, 38], which focused on the effect of different drugs (DCF, IBU) on different fish species (carp, medaka, and rainbow trout) and their organs (kidney, liver, and gonad) and did not find any tissues alterations.

The development of carp appeared to be delayed due to DCF exposure at the beginning of the early life stages test, but there was no difference after 30 days of exposure [34]. Similar trends occurred also in our experiment. After 14 days of exposure to mixture of drugs, there were all larvae in L3 stage up to concentration of 0.2 *μ*g·L^−1^. Along with increase of mixture concentration (2, 20, and 60 *μ*g·L^−1^) count of larvae of L1 and L2 stages was increased. Similarly, slightly delayed development occurred after 21 days of exposure. However, fish after 29 and 35 days of exposure had similar development.

DCF and CBZ affect fish behavior through different mechanisms. Feeding behavior of adult Japanese medaka fish was affected by exposure to CBZ and DCF, while swimming speed was altered only by exposure to CBZ [48]. The ability of hatched larvae to swim upward was affected after embryonic injection with DCF and CBZ. The number of larvae of medaka fish failing to swim upward significantly increased with the higher doses [37]. Rainbow trout affected by DCF up to 1000 *μ*g·L^−1^ showed normal behavior [38], as well as in our experiment.

## 5. Conclusion

In summary, this paper assesses subchronic toxicity of human pharmaceuticals mixture in embryos and larvae of tench by analysing mortality, hatching success, growth, early ontogeny, histopathological changes, and incidence of malformations. Our results lead us to conclude that environmental concentrations (0.02 and 0.2 *μ*g·L^−1^) of mixture of ibuprofen, diclofenac, and carbamazepine do not have an adverse effect on tench. The highest used concentration (60 *μ*g·L^−1^ for each substance) of that mixture significantly elevated mortality, delayed development of larvae, and increased occurrence of malformations. The parameters analyzed provide useful information about the effect of commonly occurred human pharmaceuticals in water environment on fish.

## Figures and Tables

**Table 1:** Concentrations of ibuprofen (IBU), diclofenac (DCF), and carbamazepine (CBZ) in surface waters.

	Surface water (dams, lakes, and rivers)					Location	Source
	Frequency (%)	Median (ng·L^−1^)	Mean (ng·L^−1^)	Min (ng·L^−1^)	Max (ng·L^−1^)		
IBU		20	20		100	Rhine river	[13]
	62	6	31.3		395	EU rivers	[14]
	96		93			Ebro river, Spain	[11]
	100		790	160	2710	Llobregat river, Spain	[15]

DCF		46	50		900	Rhine river	[13]
	83	5	17		247	EU rivers	[14]
	93		58			Ebro river, Spain	[11]
	100		2200	80	18740	Llobregat river, Spain	[15]

CBZ		110	103		640	Rhine river	[13]
	95	75	11.6		248	EU rivers	[14]
	100		55			Ebro river, Spain	[11]
	90		1070	80	3090	Llobregat river, Spain	[15]
	100	455.5				Madrid	[16]

**Table 2:** List of pharmaceuticals used in the experiment.

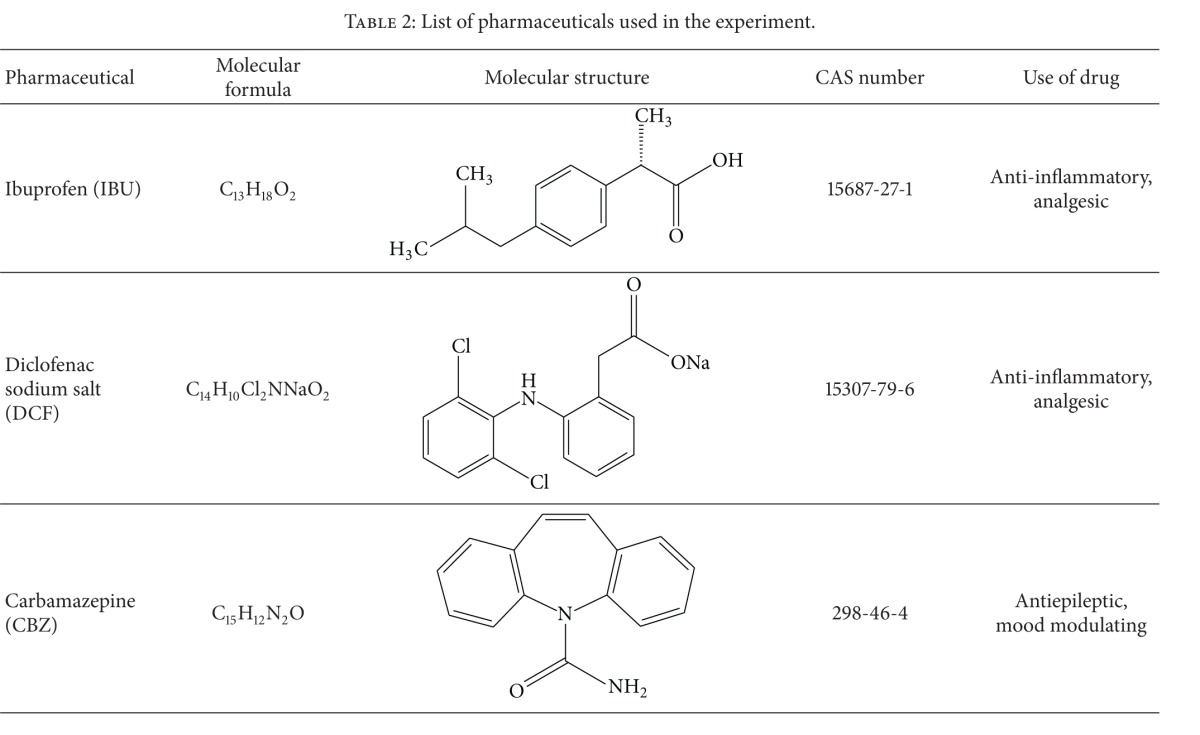

**Table 3:** Physical properties of ibuprofen (IBU), diclofenac (DCF), and carbamazepine (CBZ).

Compound	p*K*_a_	log *K*_ow_	Percentage of parent compound excreted
IBU	4.5–5.2	4.0	≤5
DCF	4.15	4.5	6–39
CBZ	13.9	2.24	≤5

**Table 4:** Average (*A*) exposure concentrations of ibuprofen (IBU), diclofenac (DCF), and carbamazepine (CBZ) ± standard error of mean (SEM) in control (C), control-solvent (CS), and experimental groups (M1–M5).

	C	CS	M1	M2	M3	M4	M5
	*A* ± SEM	*A* ± SEM	*A* ± SEM	*A* ± SEM	*A* ± SEM	*A* ± SEM	*A* ± SEM
IBU	N.d.	N.d.	0.02 ± 0.01	0.20 ± 0.05	1.66 ± 0.20	18.10 ± 0.52	56.20 ± 8.56
DCF	N.d.	N.d.	0.02 ± 0.00	0.19 ± 0.05	1.67 ± 0.17	18.30 ± 2.20	59.45 ± 9.10
CBZ	N.d.	N.d.	0.02 ± 0.01	0.18 ± 0.04	1.58 ± 0.15	18.00 ± 1.50	58.10 ± 4.51

N.d.: not detected.

**Table 5:** Average (*A*), median (M), and standard error of mean (SEM) of weight (*W*) and length (TL) parameters of tench exposed to C-control, CS-control with solvent, M1, M2, M3, M4, and M5—mixture of ibuprofen, diclofenac, and carbamazepine on concentration of 0.02, 0.2, 2, 20, and 60 *μ*g·L^−1^, respectively.

	*W*_7th day_	*W*_14th day_	*W*_21st day_	*W*_29th day_	*W*_35th day_
	*A* ± SEM (M)	*A* ± SEM (M)	*A* ± SEM (M)	*A* ± SEM (M)	*A* ± SEM (M)
C	0.95 ± 0.02 (0.90)	5.13 ± 0.12 (5.00)	13.66 ± 0.69 (12.20)	25.96 ± 1.19 (25.10)	41.98 ± 1.42 (42.10)
CS	1.00 ± 0.03 (1.00)	5.00 ± 0.10 (5.10)	16.33 ± 0.69 (15.85)	25.94 ± 1.69 (23.40)	46.35 ± 1.81 (47.00)
M1	0.99 ± 0.03 (0.95)	4.94 ± 0.18 (4.80)	16.33 ± 0.68 (15.85)	28.15 ± 2.32 (28.20)	50.18 ± 1.93 (48.10)
M2	0.99 ± 0.03 (0.95)	4.56 ± 0.19 (4.70)	15.52 ± 0.73 (14.19)	27.79 ± 1.39 (27.10)	45.74 ± 1.92 (44.40)
M3	1.02 ± 0.02 (1.00)	5.03 ± 0.15 (4.95)	16.22 ± 0.86 (14.65)	25.52 ± 1.55 (24.90)	45.95 ± 2.22 (47.50)
M4	1.03 ± 0.04 (1.00)	4.90 ± 0.30 (5.20)	13.69 ± 1.42 (15.10)	30.95 ± 2.41 (30.60)	46.72 ± 2.29 (47.60)
M5	1.09 ± 0.03 (1.10)*	3.82 ± 0.42 (4.35)*	10.12 ± 1.65 (9.60)	25.71 ± 2.87 (24.30)	62.93 ± 2.81 (68.20)**

	TL_7th day_	TL_14th day_	TL_21st day_	TL_29th day_	TL_35th day_
	*A* ± SEM (M)	*A* ± SEM (M)	*A* ± SEM (M)	*A* ± SEM (M)	*A* ± SEM (M)

C	5.14 ± 0.03 (5.12)	8.50 ± 0.06 (8.52)	11.33 ± 0.14 (11.30)	13.58 ± 0.21 (13.32)	15.70 ± 0.16 (15.89)
CS	5.18 ± 0.02 (5.18)	8.49 ± 0.07 (8.51)	11.43 ± 0.14 (11.42)	13.73 ± 0.22 (13.37)	16.13 ± 0.20 (16.30)
M1	5.09 ± 0.03 (5.13)	8.26 ± 0.14 (8.36)	11.78 ± 0.14 (11.67)	13.84 ± 0.35 (13.86)	16.40 ± 0.20 (16.55)
M2	5.26 ± 0.03 (5.27)	8.35 ± 0.12 (8.41)	11.48 ± 0.20 (11.24)	13.80 ± 0.21 (13.81)	15.95 ± 0.20 (16.08)
M3	5.18 ± 0.03 (5.16)	8.57 ± 0.07 (8.58)	11.92 ± 0.14 (11.96)	13.66 ± 0.20 (13.75)	15.97 ± 0.22 (16.10)
M4	5.27 ± 0.04 (5.32)*	8.06 ± 0.17 (8.23)	11.18 ± 0.32 (11.50)	14.22 ± 0.34 (14.22)	16.26 ± 0.23 (16.52)
M5	5.31 ± 0.02 (5.32)**	7.70 ± 0.22 (8.00)*	10.22 ± 0.42 (10.24)	13.57 ± 0.35 (13.20)	17.46 ± 0.24 (17.94)**

The asterisks indicate significant difference (*P < 0.01 and **P < 0.001).

**Table 6:** Fulton's condition factor (FCF), specific growth rate (SGR), inhibition factor (*I*), average (*A*), and standard error of mean (SEM) of C-control, CS-control with solvent, M1, M2, M3, M4, and M5—mixture of ibuprofen, diclofenac, and carbamazepine on concentration of 0.02, 0.2, 2, 20, and 60 *μ*g·L^−1^, respectively.

	C	CS	M1	M2	M3	M4	M5
	*A* ± SEM	*A* ± SEM	*A* ± SEM	*A* ± SEM	*A* ± SEM	*A* ± SEM	*A* ± SEM
FCF_35_	1.07 ± 0.01	1.07 ± 0.01	1.11 ± 0.01	1.12 ± 0.02	1.07 ± 0.02	1.06 ± 0.01	1.15 ± 0.02**
SGR	13.52	13.70	14.01	13.68	13.59	13.62	14.49
*I* (%)	1.31		−2.26	0.15	0.80	0.58	−5.77

The asterisks indicate significant difference (*P < 0.01 and **P < 0.001).
